# The Chronic Indigence of Our Hospitals, with Some Suggestions for Its Remedy

**Published:** 1892-04-16

**Authors:** B. Burford Rawlings


### The Institutional Workshop.

### THE CHRONIC INDIGENCE OF OUR HOSPITALS, WITH SOME SUGGESTIONS FOR ITS REMEDY.

By B. Burford Rawlings.

### III. THE MATERIAL DEFICIT.

Curiously enough, even the best informed of speakers and writers appear to have limited their attention to the difference between the expenditure and the receipts, as though the failure to wind up the yearly accounts without a balance on the wrong side constituted everything it is needful to consider in this connection. Really, the difference between what is received and what is spent is a mere tributary to the volume of deficiency, which is not one of income only, but also of capital. A grave charge against our hospital system might be framed in reference to the inconvenient and possibly insanitary buildings in which some of the work is carried on, the defective provision for convalescents, and the partial or entire neglect of certain sections of sufferers.

If as a metropolitan community we were seriously bent upon producing a hospital system worthy of science and philanthropy alike, it is certain we should be meditating a large initial expenditure upon land and buildings. Yet where is the hospital whose managers have done their best to substitute suitable for unsuitable buildings, wholly or in part, without being assailed for their extravagance and without losing some of their most valued friends, who decline to sanction an outlay upon "bricks and mortar?"

At this moment we have University College Hospital, an institution with a great reputation, both philanthropic and educational, lodged in a building worthy of the dark ages. For many years its authorities have been alive to the importance of raising a suitable structure, and there is reason to believe that their experience has coincided with that of other hospitals which have been desirous of proper equipment. As the disabilities of hospitals are far more conspicuous than their powers, they are at the mercy of every neighbour who would drive a hard bargain, and whereas a railway company, or a local board may obtain the power to acquire property compulsorily, upon payment of a fair compensation, the hospital must consent to pay anything that is demanded, however exorbitant, or submit to being stopped altogether from carrying out a great public work. Nine years were once consumed before a hospital could obtain possession of a single house, and it is likely that to obtain a sufficient site in London for a large hospital would require negotiations extending beyond the term of a natural life and an expenditure of at least four times the value of the property required. It is only reasonable to contend that hospitals should be granted the same powers—to be exercised under the same control—as other organisations working for the public benefit and convenience.

The deficiency of accommodation is naturally even more palpable and distressing. It is nobody's business to determine what the sum total of hospital work ought to be, and how much of it is done, overdone, or neglected. There is no attempt to adapt the provision to the requirements, or to make it grow with the wants of an increasing population. It must not be forgotten that rate-supported sick asylums have been erected, but it is not a disparagement of their work to say that it no more relieves the voluntary hospitals than the work of the old workhouse infirmaries does, for the reason that neither in the one case nor the other is there a visiting staff or an approach to the scientific standard reached by the voluntary institutions. Moreover, they confer the taint of pauperism. Some hospitals are more hardly pressed than others, but the refusal of strictly suitable applications because there is no room is a frequent occurrence at almost all hospitals; at some it is a daily incident. Imagine the names of sick people placed upon a rotation list to be admitted in turn, after weeks or months of waiting! Yet the hospital concerned is not to be condemned. It may be doing its duty bravely and well, and its supporters are entitled to the gratitude of all men. The rejected ones are not ready to become paupers, they would not obtain what they want though they were. If we inquire what their fate is, the answer must be disquieting to sensitive minds; it is fatal to the contention that the present system does all that is required of it and it is a forcible protest against continued apathy. When a would-be patient is told there is no bed, that he must wait his turn, or is refused outright and goes back to his home to suffer inadequately tended, or it may be, to pay the full penalty of his involuntary dependence upon the voluntary system by dying, it is little consolation to him or to his friends to reflect, if they be capable of so great philosophy, that they are sacrificed at the shrine of a glorious sentiment. They would doubtless prefer that practical provision should be made, even though less philanthropic in origin, for the relief of everyday and but too familiar suffering.

It only aggravates the case to be told, even supposing it to be true, that the total money subscribed is sufficient for all purposes, when personal experience shows the inadequacy of the provision for particular patients. Without doubt there is a great waste of resources owing to the difficulties in the way of raising funds, and the absorption of a portion of them by redundant institutions, but whether the saving which might be effected by any method of repression is sufficient to allow of adequate provision for all classes of patients remains to be seen. The total expenditure on begging account is probably equal to the cost of maintaining 600 or 700 beds; but although the outlay under this heading might be sensibly lessened if certain efforts in unison, such as will be presently suggested were adopted, it is certain that any system which includes independent support for the several institutions must always demand a considerable outlay upon the issue and presentment of individual appeals.

The voluntary hospitals contain a total of upwards of 7,000 beds, and the question is not only whether this number is sufficient in the aggregate for the sick poor, other than paupers, of a population of five millions plus the many country patients attracted by the reputation of the metropolitan hospitals, but also whether the different classes of sufferers are all equally and adequately provided for. In making the calculations we must take into consideration the vast industrial enterprises centred in the capital, with the many dangers to the work-people and others they involve, our docks, railways, breweries, and the like, together with that prolific source of mishap, the traffic of the congested streets. Necessarily a very large proportion of these beds are required for cases of accident, and to that extent the provision for cases of ordinary and epidemical disease is lessened. Then again, as our voluntary hospitals are schools of medicine, the demands of science, legitimate enough in their way, must more or less conflict with the therapeutical side of hospital work, and when the beds are too few for all requirements there is almost necessarily a tendency towards a selection made in the interests of the clinique rather than of the patients, thus diminishing still further the provision for the direct relief of suffering. Beyond all this, we must remember the very important fact that the 7,000 beds are divided among 70 or 80 different institutions, many of which limit their work to one sex, to children, or to a single class of malady, while from even the general hospitals, certain sets of sufferers are excluded.

Considerable difficulties stand in the way of making an accurate calculation of the number of beds available for London patients in convalescent homes, but that the provision is deficient is generally agreed, and on all sides we hear of sickly and debilitated persons who recently have been inmates of the hospitals and to whom fresh air would be a veritable elixir of life, waiting day by day and week by week in the hope of admission to one or another of the homes supposed to provide for the convalescents of our great City.

When we consider what a boon and a restorer, far transcending all drugs, is change of scene and air, it seems worthy of discussion whether we should not supply the Hospital Physician with far greater facilities than he now possesses for prescribing it; whether, in fact, every hospital ought not to possess as a matter of course country and sea-de homes under its own management just as it now dispenses for itself every other kind of medicine.

A striking illustration of the unsatisfactory nature of an arrangement under which hospital patients needing change must seek the good offices of an independent institution was afforded when a well-known convalescent institution — now happily conducted upon more righteous principles—refused to allow a hospital to subscribe upon the ground that the latter would want to make use of its privileges! When it is stated that a payment of 7s. per week is required of each inmate in addition to the production of a letter, making a total weekly contribution of 10s. 6d., which is almost, if not quite an equivalent to the cost of his food, the unreason of the refusal becomes still more apparent. In the more enlightened future, which we venture to think hospital work has before it, we may be sure that the physicians will be enabled to order "change" like any other medicine, and convalescent wards will be considered as a part of the needful equipment of every medical institution.

As an example of inadequacy of provision, it may be stated that deaths from diseases of the nervous system are computed to exceed a yearly total of 100,000 in England and Wales alone, while for the poor of the vast array of sufferers represented by these figures, and who are virtually excluded from general hospitals, there is no provision outside London, and there the number of beds available in special hospitals is no more than 230. As a consequence, for every patient admitted into the chief institution devoted to these maladies—the National Hospital, in Queen Square—three or four others equally deserving, and in almost equally urgent case, must be refused. We believe there is always a list of waiting patients at the several hospitals for consumption, and a similar statement has lately gone forth in regard to the Ormond Street Hospital for Children. As many other hospitals for children exist, and nearly all hospitals contain wards for children, this fact tells strongly in favour of the presumption that the accommodation for in-patients is generally insufficient.

Some forms of suffering are overlooked altogether. For that class of brain disease of which melancholia in its earlier stages may be taken as an example, where the subject is threatened with insanity, yet whom no two medical men would dare to certify as insane—for those who stand upon the borderland which separates reason from lunacy, and by timely care may be saved from a fate worse than death—persons, in short, whose sufferings cry for relief as loudly as any upon the face of the earth, for these no helping hand is stretched anywhere. They are quite unfit to be placed among ordinary sick folk, so cannot share the advantages of existing hospitals, and in the absence of special provision, must be left to qualify for the asylum. It may be remembered that a proposal was brought forward at the London County Council by Mr. Brudenell Carter to establish a small hospital for the active treatment and study of diseases of the brain involving insanity. This proposal was rejected, but had the result been different, the want just indicated, would not have been supplied. Mr. Carter's proposition was to provide scientifically for actual lunacy and, moreover, the patients were to be paupers. But in the one case as in the other, our authorities are content to do nothing, and neglectful of the splendid promises of advancing science, they seem to exclaim with incredulity "Who can minister to a mind diseased?"

One thing, however, is certain in this connection. The very worst way to set about remedying the deficiency of provision for particular classes of patients is the starting of fresh hospitals by irresponsible people. No more fatal course could be pursued than to add to the number of hungry and competing institutions. What is wanted is consolidation, and where possible, amalgamation. Few people perhaps fully realise the moral effect which the undignified scrambling for crumbs of help has upon many of the onlookers. It is at once the strength and weakness of our present method that whatever an institutions obtains, it owes, as we have seen, to the favour of individual supporters whose compassion has been aroused and whose assistance is seldom adjusted by a nice sense of proportion. As a consequence, donations and bequests are sometimes made with bewildering disregard of circumstances, and petty societies, whose appeals are rich in that *suggestio falsi* which is responsible for a great part of the distrust and indifference charitable efforts often encounter, because most people now feel little hesitation about dividing statements concerning philanthropic work into more or less unequal portions of truth and fiction—obtain by sheer audacity the help for which better organizations may look in vain.

It has been argued that the liberty to establish a hospital and to solicit subscriptions ought not to be interfered with, because such support as is accorded is voluntarily given, and people may do as they like with their own. Surely there is a fallacy here. The employment of a quack doctor is a voluntary act, and the money paid him is the victim's own, but nevertheless quacks are not allowed to practise, and if they do it is at their peril. The law protects the public against impositions of various kinds, why not then against sham or useless hospitals? Besides, the patients have some claim to consideration. A physician of a small special hospital has been known to fail in his examination for a degree chiefly by reason of his ignorance in respect of the very maladies he had adopted as his speciality. Money is often sent to particular places which would be withheld or sent elsewhere if the actual facts were known; while there is reason to suspect that confusion is in some cases deliberately fostered. A case has occurred in which a large amount was forwarded to an institution—not a hospital by the way—whose never useful existence had terminated, and whose chief promoter had been prosecuted, and in another instance an executor entrusted with the distribution of a quarter of a million sterling frankly admitted that £1,000 had been paid to a hospital for which it was not intended.

As a very small measure of reform, it might be made compulsory upon every society which appeals for funds to publish a plain statement of its work, properly vouched for, and no imitation of name should be allowed. At present an unincorporated hospital may have its full title appropriated without remedy. Confusion, even when the genuine character of an institution is not in question, is still regrettable, and may lead to serious, if occasionally ludicrous errors. Witness the gift a few months ago of eighty pheasants to a hospital containing twenty patients. The illustration is not less effective, because in that case the hospital, though small, was irreproachable.

Again, we read of juries censuring thoughtless employers who provide no adequate means of exit for their fire-bound workpeople. What have we to say of the license accorded to every one of us to open any building whatever, suitable or unsuitable, safe or unsafe, for hospital purposes, and to fill its several floors with sick and helpless persons? With their want of space, thin walls, wooden staircases, and tortuous passages, many of our "adapted" hospitals and even some more pretentious, are admirably contrived to make escape hopeless in the event of the terrible calamity of fire.

Great moral advantages would be gained were means devised which, without interfering with legitimate work, would be capable of purging charity of its excesses. Beyond this, we might look for a positive increase in material usefulness. As the number of hospitals lessened, the provision for the sick would increase and expenditure relatively, if not actually, would diminish. Those who will, may regard this as a paradox; it is nothing but plain truth.

